# A mechanistic view on the aging human skin through ex vivo layer-by-layer analysis of mechanics and microstructure of facial and mammary dermis

**DOI:** 10.1038/s41598-022-04767-1

**Published:** 2022-01-17

**Authors:** Barbara Lynch, Hervé Pageon, Heiva Le Blay, Sébastien Brizion, Philippe Bastien, Thomas Bornschlögl, Yegor Domanov

**Affiliations:** L’Oréal Research and Innovation, Advanced Research, Aulnay-sous-Bois, France

**Keywords:** Tissues, Ageing

## Abstract

Age-related changes in skin mechanics have a major impact on the aesthetic perception of skin. The link between skin microstructure and mechanics is crucial for therapeutic and cosmetic applications as it bridges the micro- and the macro-scale. While our perception is governed by visual and tactile changes at the macroscopic scale, it is the microscopic scale (molecular assemblies, cells) that is targeted by topical treatments including active compounds and energies. We report here a large dataset on freshly excised human skin, and in particular facial skin highly relevant for cosmetics and aesthetic procedures. Detailed layer-by-layer mechanical analysis revealed significant age-dependent decrease in stiffness and elastic recoil of full-thickness skin from two different anatomical areas. In mammary skin, we found that the onset of mechanical degradation was earlier in the superficial papillary layer than in the deeper, reticular dermis. These mechanical data are linked with microstructural alterations observed in the collagen and elastic networks using staining and advanced imaging approaches. Our data suggest that with ageing, the earliest microstructural and mechanical changes occur in the top-most layers of dermis/skin and then propagate deeper, providing an opportunity for preventive topical treatments acting at the level of papillary dermis.

## Introduction

Age-related changes in skin mechanics are thought to contribute for a large part in the degraded perception of skin appearance and feel, e.g. wrinkles, loss of firmness and elastic rebound. The knowledge of skin's mechanical properties is of great importance in many clinical and cosmetic applications, including age management treatments by the topical application of active molecules, the use of energy treatments (e.g. mechanical, radiofrequency or laser stimulation), surgeries, skin tissue generation for burn injury treatment, etc. A precise knowledge of the link between skin microstructure and mechanical properties in relationship with age is crucial for therapeutic and cosmetic applications as it bridges the microscopic scale (molecules and their assemblies) with the tissue properties at macroscopic scale. While the perception of patient or consumer is governed by the changes at the macroscopic scale, it is the microscopic scale that is targeted by topical treatments (active compounds or energies). There is in particular a strong interest for locating the onset of age-related degradation in the dermis, as this will inform future strategies to best delay and counteract the earliest effects of age and prevent further damage. If the changes start to occur in the deepest layers of the skin, only very invasive techniques, through esthetic surgery, will make a true difference. On the other hand, if the changes occur first in the uppermost layers of the dermis, we may have an opportunity to correct the first signs of aging through topically applied creams in a preventive action.

Skin is a multilayered structure: the outermost part is the epidermis, a layer of stratified cells, then comes the dermis and the deepest layer is the hypodermis. The dermis also happens to be the thickest and most important layer for mechanics. It consists mostly of collagen and elastin fibers embedded in a host gel-like structure^1^. The dermis is further divided into two layers with different morphological features, the upper, papillary dermis and the deeper, reticular dermis. In the reticular dermis, the fibers are generally thicker than in the papillary dermis and often bundled together. The papillary dermis is much thinner than the reticular dermis: the thickness of the human papillary and reticular dermis varies, with age, between 60–120 µm and 1000–4000 µm respectively^2^.

Skin ageing from the microstructural point of view has been studied mainly through the observation of histological cuts with various staining. Classical histological features of ageing skin include a reduction in epidermal thickness, flattening of the dermo-epidermal junction and alteration of dermal structures^3^. The dermis is particularly affected by ageing as it has a low turn-over compared to other skin layers: while the epidermis is renewed in about a month time^4^, the dermis renewal rate is estimated between 11 years and lifespan^5^ (e.g. skin collagen half-life is 15 years^6.^ The most noticeable microstructural modifications of the dermis with age^5^ have been reported as a decrease of collagen^2^ and elastin contents^7^, an increase of collagen cross-linking^6^, and a deterioration of proteoglycans^8^.

Skin ageing can result from both the passage of time (intrinsic) and from cumulative exposure to external factors (extrinsic), such as UV light in photoexposed areas. The severity of the resulting microstructural changes, age of onset and alteration rate are known to be exacerbated in photoexposed skin^5,7^.

Most experimental studies on the age-related changes in the mechanical properties of skin have been carried out on human skin in vivo, using torsion or suction devices^9–11^. Ex vivo experiments remain marginal, due to difficult access to appropriate samples^12,13^. For our problematic of locating the onset of the age-related changes in skin mechanics and linking them with specific alterations of the microstructure of the dermis, we need to be able to access specific layers individually, both for mechanical characterization and microstructural observations. Ex vivo skin is in this regard the most suited solution, as it combines mechanical and structural properties close to in vivo skin with relatively easy access to specific layers. In the study presented here, we studied the impact of age on the mechanical properties of ex vivo skin from a large number of human donors, from both photoprotected (mammary) and photoexposed (cheek) skin. To the best of our knowledge, it is the first study reporting both mechanical and microstructural results on ex vivo facial skin, which is highly relevant as this is where the modifications of skin mechanics are the most drastic and most visible for patient or consumer. We also studied the mechanics of isolated dermal layers to locate the onset of the age-related changes in the dermis and to help better define age-management strategies. Finally, through microstructural observations, we propose to identify relevant biological targets suspected to be at the origin of the observed age-related changes.

## Results

### Age-related decrease in stiffness and elasticity in full thickness skin

**Figure 1:**
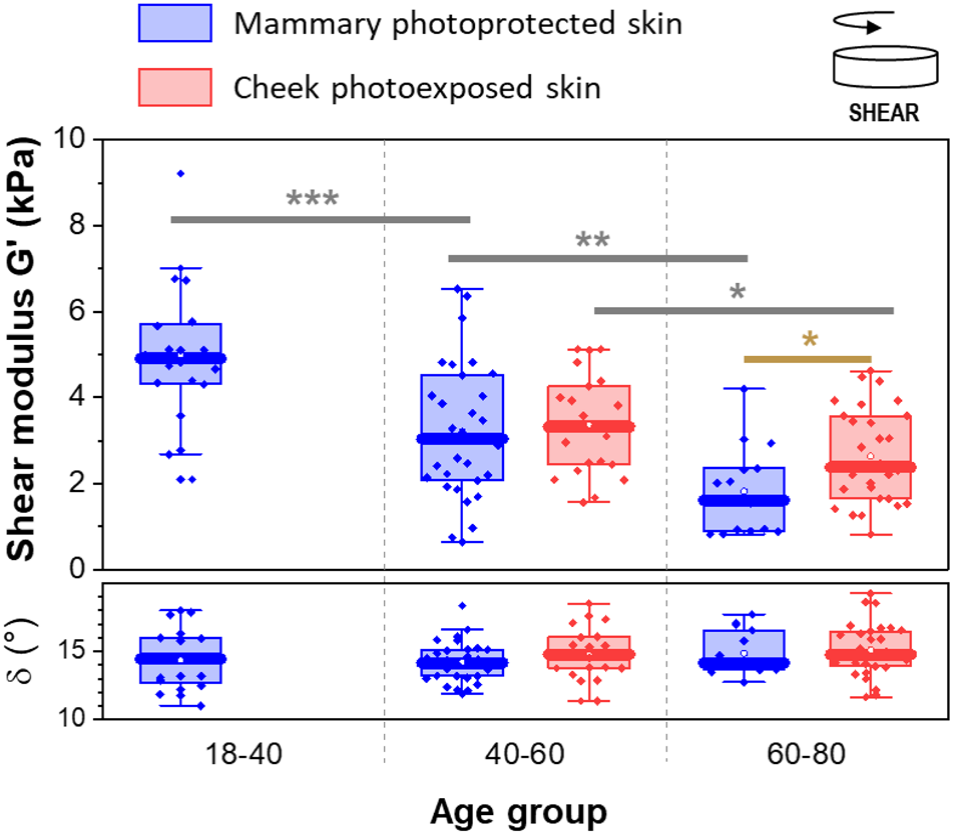
Storage modulus G′ (upper panel) and Phase angle δ (lower panel) measured by shear deformation as a function of age group, for photoprotected mammary (blue) and photoexposed cheek (red) full-thickness skin. One dot represents one skin sample. Multiple samples were obtained for each donor. *p < 0.05, **p < 0.01, ***p < 0.001.

As we had access to a wide range of fresh full-thickness human skin samples, we could systematically study the biomechanical properties of skin as a function of donor age. As presented in Fig. 1, strong alterations with age were found in the mechanical properties of the full-thickness skin.

A decrease in stiffness (storage modulus G′) was observed for shear deformations at small strains, on full thickness skin of both mammary (photoprotected) and cheek (photoexposed) skin (Fig. 1, upper panel). Mammary skin in the oldest age group was found to be almost 3 times softer than in the youngest group (median G′ values were 4.9 kPa and 1.6 kPa, respectively). This reduction was progressive throughout the lifetime. Similarly, a significant decrease of stiffness was observed from mid-aged to older facial skin as well, from a median G′ value of 3.3 kPa to 2.4 kPa respectively, suggesting a similar trend of progressive degradation. Unfortunately, as facial skin was obtained as surgical waste from lifting surgeries, no young skin from facial origin could be obtained to observe the onset of the trend.

Furthermore, cheek skin was found to be stiffer than mammary skin for the oldest age group, with a median G′ value of 2.4 kPa compared to 1.6 kPa respectively. This could be due to the impact of photoexposition^14^ and/or to the different anatomical locations, as we know that skin mechanical properties depend strongly on body location^15,16^.

The phase angle parameter (δ) can be used to evaluate the relative importance of elastic/viscous behaviors. No changes in the phase angle were seen with age or as a function of photoexposition/anatomical location (Fig. 1, lower panel).

**Figure 2:**
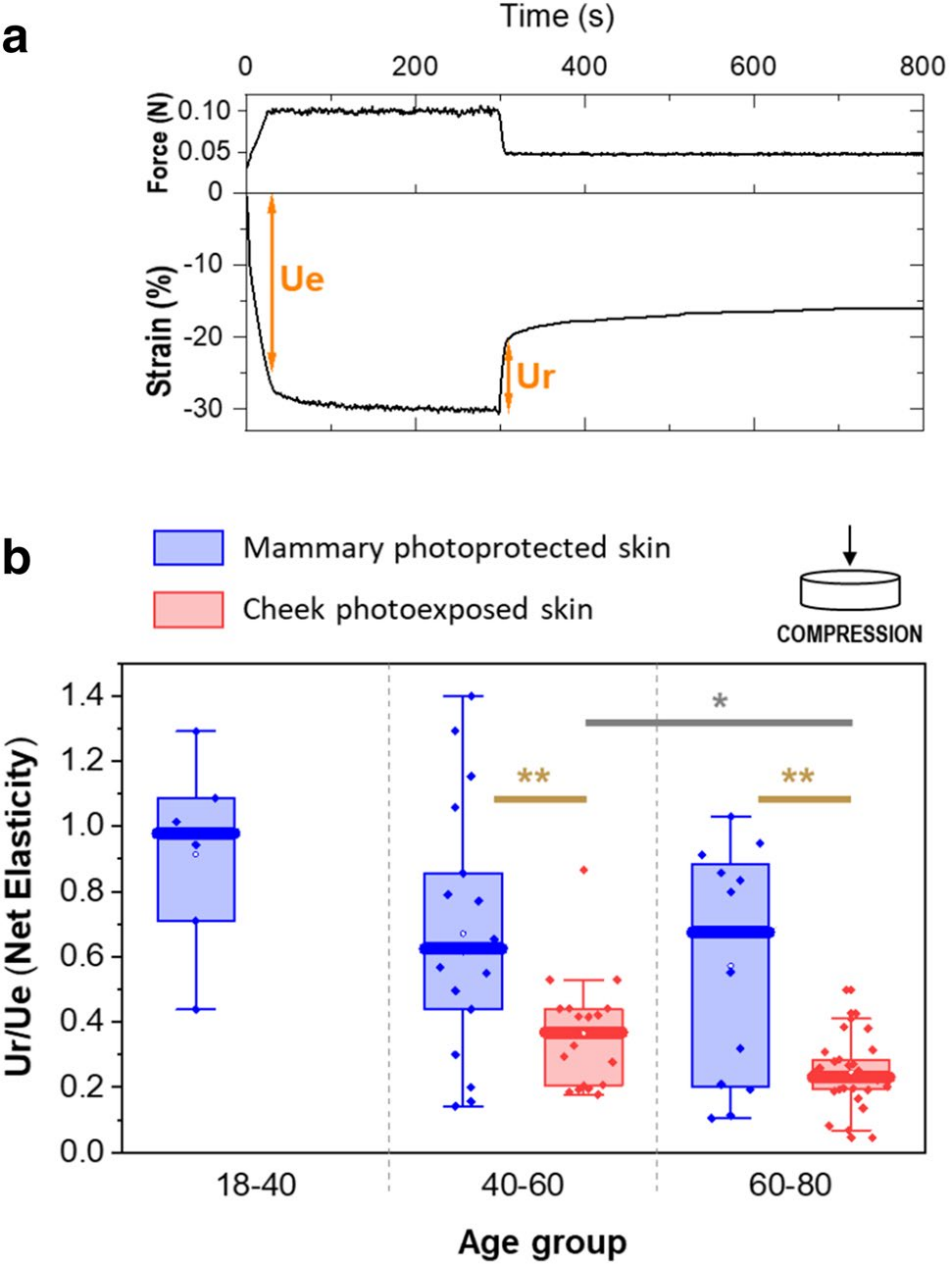
Elastic recoil of full-thickness skin after compressive deformation perpendicular to skin surface. (**a**) Time course of stress (upper panel) and strain (lower panel) in a typical compression experiment, together with the definition of parameters Ue and Ur (see “Materials and methods” for more details). (**b**) Net elasticity (Ur/Ue) of photoprotected mammary (blue) and photoexposed cheek (red) skin as a function of age group. One dot represents one skin sample. Multiple samples were obtained for each donor. *p < 0.05, **p < 0.01.

In addition to profound modifications in stiffness, the elastic response (recovery following the removal of compressive deformation) was also found to be dependent of age and anatomical location. As shown in Fig. 2, a significant decrease was found in cheek skin between the mid-aged and the oldest groups for the net elasticity parameter, which captures the ratio of the fast recovery Ur to the fast deformation Ue in compression mode. Ur/Ue fell from 0.4 to 0.2. These observations were confirmed also on other elasticity parameters (gross elasticity and biological elasticity, see Supplementary Fig. S1).

A strong decrease in elasticity was observed in the 40–60 and 60–80 age groups when comparing mammary skin and cheek skin, as Ur/Ue decreased by 40 and 70%, respectively. In other words, aged facial skin was less capable to spring back after compressive deformation as compared to aged mammary skin, and this gap in recoil properties appears to widen with age. This difference could again be attributed either to difference in photoexposition, difference in body site or both.

The modifications of the skin viscoelastic behavior were visible on the elasticity parameters in compression under large deformations, but not observed on the phase angle in shear at small deformations, which can be attributed to different strain types and extent.

No obvious differences in mechanics were found for the skin originating from male donors (2 donors) versus female donors (20 donors) in cheek skin.

### In mammary skin, age-related changes in mechanics occur first in the papillary layer

Papillary and reticular dermis sublayers are known to have distinct structures, chemical compositions and biological functions^17,18^. Considering these differences, we attempted to characterize the biomechanical properties of the two sublayers individually on photoprotected mammary skin. For this purpose, we isolated each dermal sublayer using a razor cutting tool (keratome) and acquired the mechanical properties of each sublayer individually. Samples large enough for rheometer experiments were obtained in the papillary sublayer for 15 donors and reticular sublayer for all 17 donors (all female donors). The sublayer characterization was not possible on facial skin due to small sample size.

**Figure 3:**
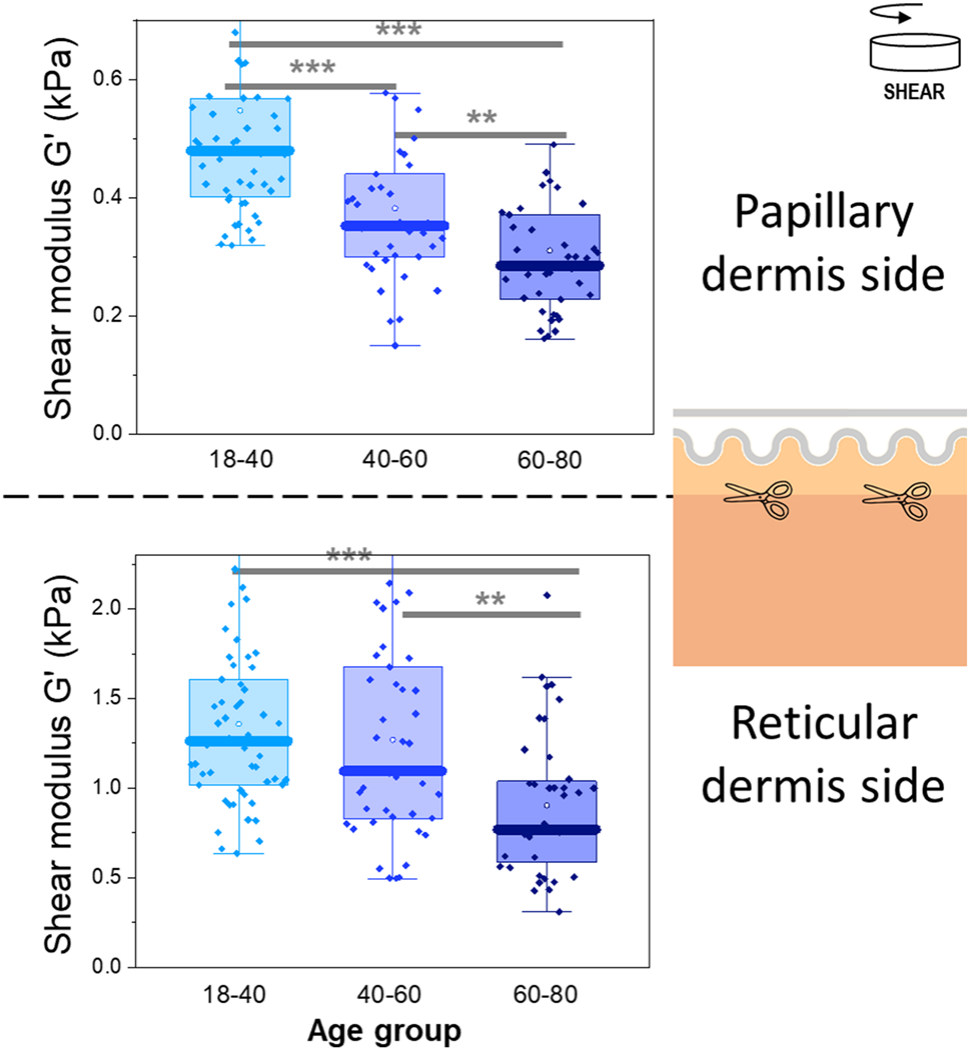
Storage modulus G′ (kPa) as a function of age group for the papillary dermis (top) and reticular dermis (bottom) for photoprotected mammary skin. The epidermis layer has been removed prior to measurements. One dot represents one skin sample. Multiple samples were obtained for each donor. **p < 0.01, ***p < 0.001.

Firstly, we can see that, as shown in Fig. 3, the age-related decrease in stiffness observed on mammary full thickness skin was also observed on isolated dermal layers, both papillary and reticular. The decrease in median G′ was about 40% from the youngest to the oldest age groups, in both layers. The decline of mechanical properties was found to occur earlier in the superficial papillary layer than in the deep reticular dermis. A very significant decrease in stiffness can already be observed between the first two age groups (18–40 and 40–60) in papillary dermis, while the decrease in the reticular dermis was only observed between the 40–60 and 60–80 age groups. No associated change in the phase angle was observed (Supplementary Fig. S2).

Secondly, we observed that the papillary dermis is significantly softer than the reticular dermis. Indeed, we noted that for 14 out of the 15 donors for which both reticular and papillary sublayers could be characterized (Fig. 3), the reticular layer was found to be on average 3 times stiffer than the papillary sublayer (Supplementary Fig. S3). This difference cannot be attributed to the difference in thickness between the two layers (the reticular dermis being much thicker than the papillary dermis) as the storage modulus measured is an intrinsic material property independent of dimensions (i.e., sample thickness is taken into account when calculating the modulus). No evolution of the mean reticular stiffness over mean papillary stiffness ratio was found with age (Supplementary Fig. S3). No difference in relative elastic/viscous behavior (phase angle) was found between papillary and reticular layers (Supplementary Fig. S2).

Out of the 15 donors for which both papillary and reticular layers were characterized, 13 were also characterized with full thickness skin samples (2 not characterized due to time constraints). We have noticed that for 12 out of these 13 samples, the storage modulus of full thickness skin was larger than that of each of the two dermal layers, papillary or reticular. Specifically, we observed: G′Full thickness > G′reticular > G′papillary. Full thickness skin was about 3 times stiffer than the reticular dermis sublayer.

Such layer-by-layer mechanical analysis was only possible on mammary skin as the samples had sufficient size for keratome splitting, whereas facial lifting samples were too small. Other approaches will be necessary to confirm if the above findings can be generalized to other anatomical zones, including facial skin.

### Microstructural targets likely to modulate skin mechanical properties

#### Histological observations

For all donors, histological cross-sections of full thickness skin samples were performed, with 5 different stainings to characterize the overall morphology (HES), collagen network (Sirius Red), collagen quality (Herovici), elastic network (Orcein) and collagen-proteoglycan morphology (Mowry Van Gieson). The main known chronological age-related characteristics^5^ were also observed in our quantitative histological analysis (Supplementary Figs. S4, S5, S6). Briefly, the epidermis becomes thinner and the dermo-epidermal junction flattens. The dermis becomes atrophic, the number and diameter of collagen fiber bundles decrease, the elastic network perpendicular to the epidermis disappears and the deeper network deteriorates. These observations are exacerbated with UV photoexposure, and we also observe an epidermal atrophy and fragmentation of collagen and elastic fibers in the dermis, due to the accumulation of nonfunctional elastin material, which forms aggregates and is known as elastosis^5^.

Our histological observations show that the skin samples originating from mammary explants and those from the cheek differ from both compositional and morphological point of views. We were able to observe differences in the thickness of the entire skin, in the location of the fatty tissue, in the number of hairs and glands when we compare these two distinct anatomical sites.

**Figure 4:**
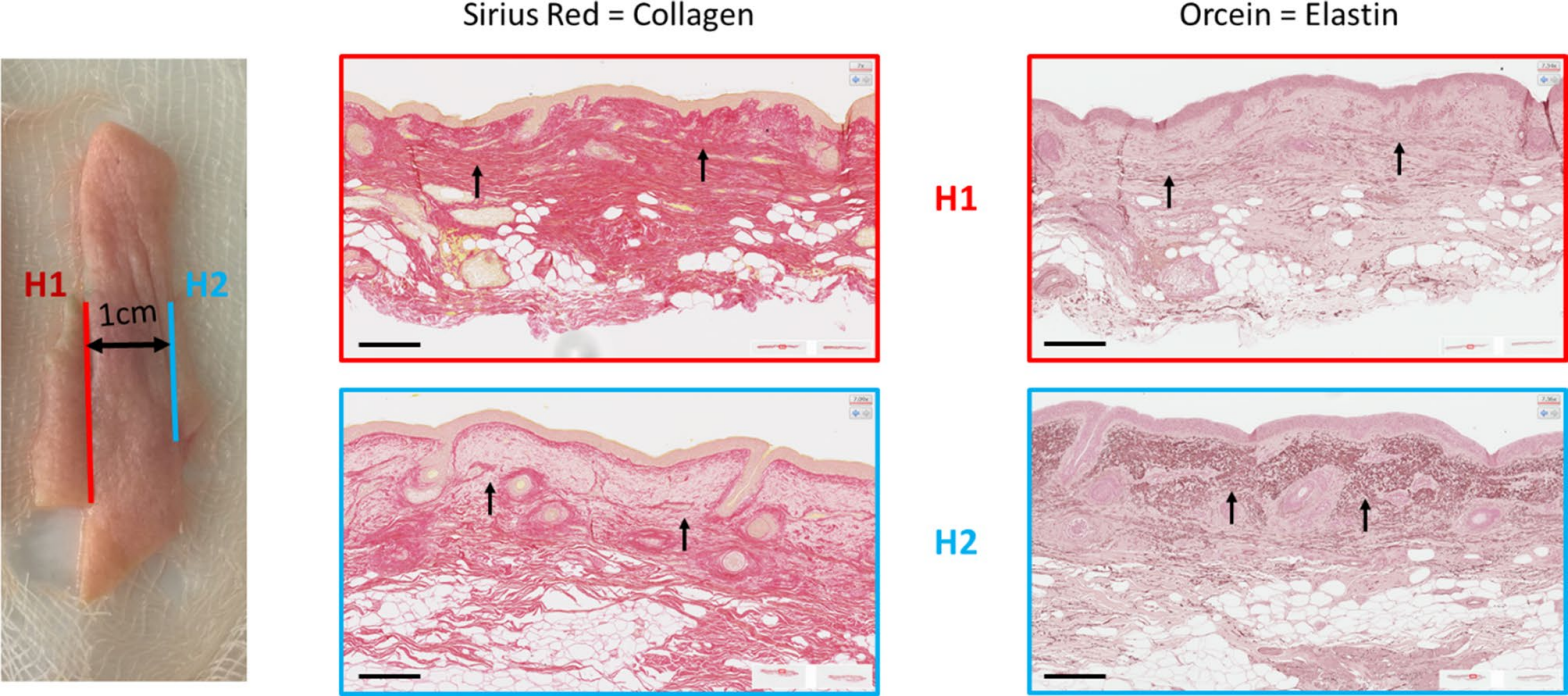
Sirius Red (left) and Orcein (right) stainings of histological cuts located 1 cm apart (× 10 magnification) on cheek skin. Arrows indicate the zone where the modification of the collagen and elastic network undergoes the most observable modifications. Scale bar is 250 µm.

More specifically for the cheek, Fig. 4 illustrates this with two histological cuts that were taken approximately 1 cm apart on the same facial skin explant. In histological cut H1, the collagen network seems preserved throughout the thickness of the cut (Sirius Red staining, left), and no elastotic material is visible (Orcein, right), while in histological cut H2 the tissue looks much more damaged: the collagen network is strongly degraded throughout the dermis and even more in the papillary dermis, an area which corresponds to the accumulation of elastotic material as indicated by the arrows.

This structural heterogeneity of facial skin becomes greater with age, as can be seen from histological sections and as a marked increase of dispersion in elastin and collagen quantification (Supplementary Fig. S4).

**Figure 5:**
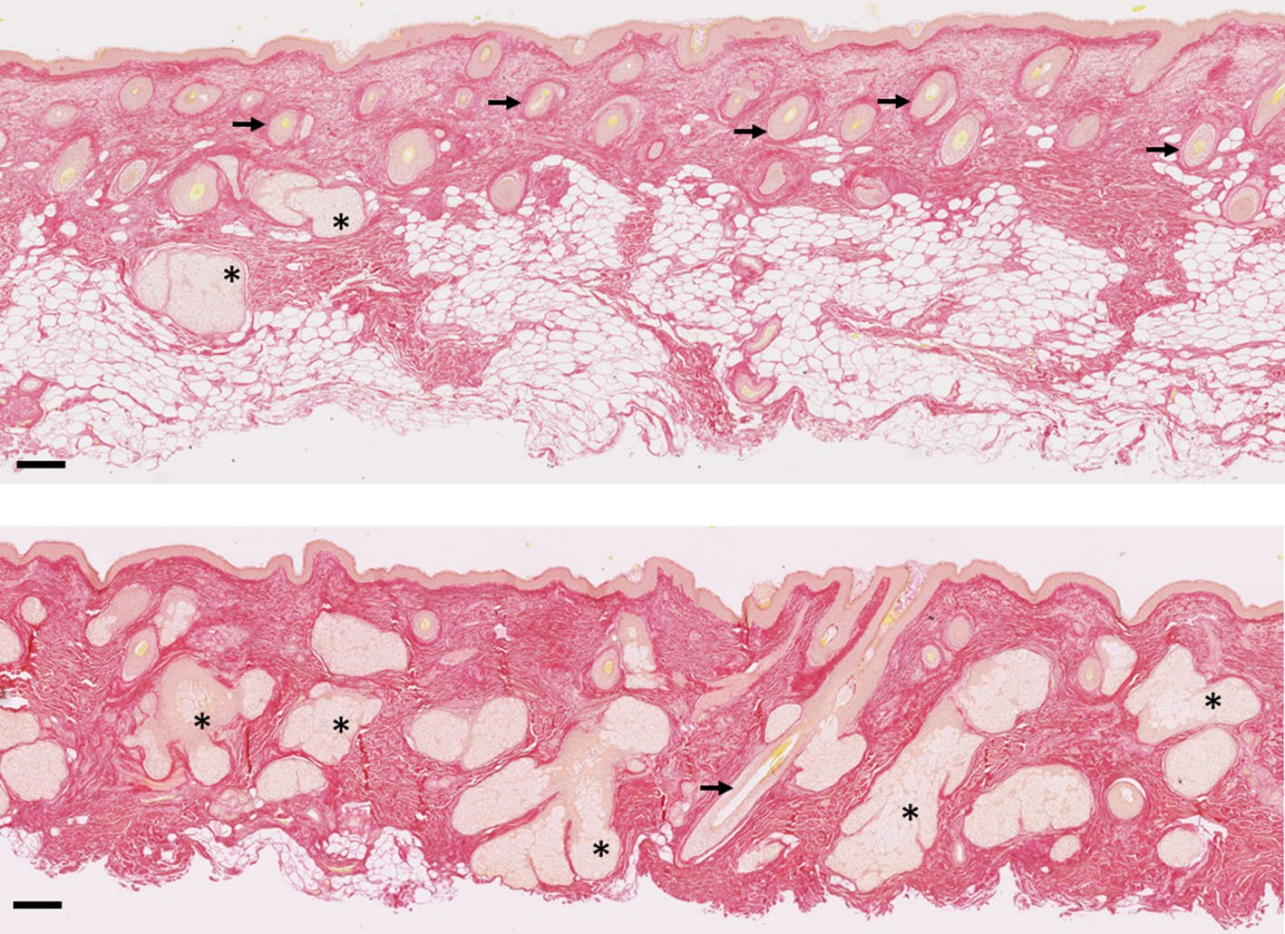
Sirius Red (collagen) stainings for two samples from photoexposed face area showing a large proportion of hair follicles (arrows) and glands (asterisks). Scale bar is 150 µm.

Furthermore, in face skin, the volume occupied by fat, glands and hair follicles was found to be variable and sometimes extremely large, as shown in Fig. 5. This aspect is usually completely left out of in vitro and in silico models, whereas it is likely to significantly affect the mechanical properties of the whole tissue. These features of facial skin are in stark contrast with mammary skin studied here and the skin structure in other “classical” anatomical locations as reported in the literature.

**Figure 6:**
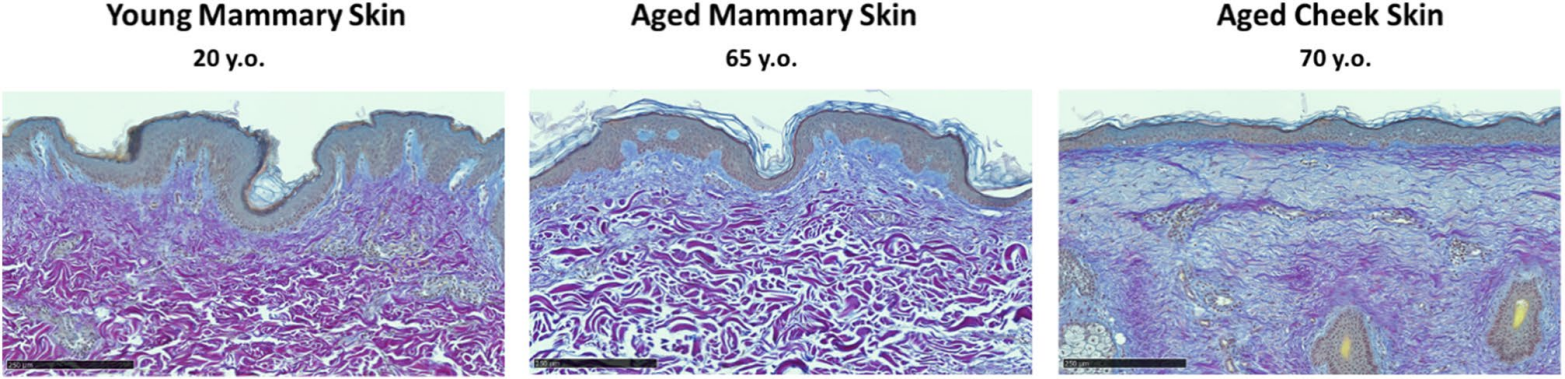
Herovici stains of young mammary skin (left), aged mammary skin (middle) and aged cheek skin (right). Purple collagen is considered intact, while collagen stained blue is degraded. Scale bar is 250 µm.

Collagen was found to be more degraded with age, and as expected the degradation was more pronounced in photoexposed skin. This is illustrated in Fig. 6, presenting Herovici stains of young mammary skin (left), aged mammary skin (middle) and aged cheek skin (right). In young mammary skin, very little collagen is stained blue (degraded). In aged mammary skin, collagen is stained blue in the papillary dermis while it remains purple (intact) in the reticular dermis. We observed that the non-stained “empty” spaces between collagen bundles increased with age. Finally, in aged cheek skin, most of the collagen in the dermis is stained blue, which means it has been degraded completely by both intrinsic and extrinsic ageing.

Histological observations have also shown a strong drop of the thickness of epidermis between the groups 18–40 and 40–60 but not after (no change between 40–60 and 60–80) (Supplementary Fig. S5). In vivo experiments have shown that the epidermis gets thinner with age, corroborating our histological observations^19^.

#### Multiphoton observations

Multiphoton images were obtained both in plane of the skin by imaging optically-clarified samples throughout the whole tissue thickness and in cross-sections. This technique of optical clarification provides unprecedented quality of multiphoton imaging of deeper dermal layers, which is largely inaccessible in transcutaneous imaging mode used in vivo. The multiphoton microscope allows us to access two signals: the Second Harmonic Generation signal (SHG), which is specific to fibrillary collagen and the Bi-photon Fluorescence signal (2PEF), which is generated by elastic fibers, cells, hairs, and to a lower extent collagen.

**Figure 7:**
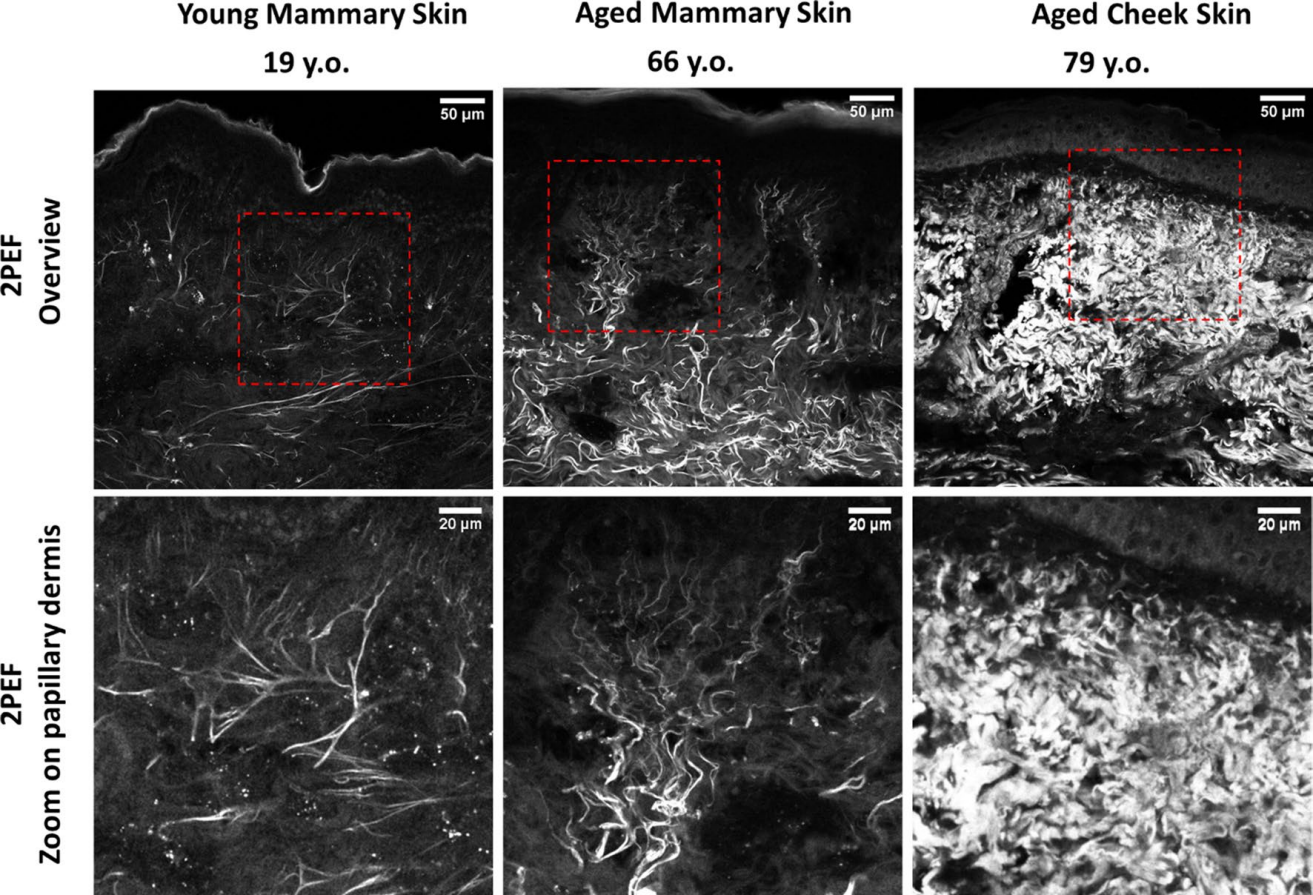
Two-photon-excited fluorescence (2PEF) images revealing elastic fibers, cells, glands in cross-sections of young mammary skin (19 years old), old mammary skin (66 years old) and old cheek skin (79 years old) observed in multiphoton microscopy. Top images are overview and lower images are zoomed on the papillary area.

Firstly, multiphoton observations confirmed, but with finer details, the morphological differences quantified in histological cuts. In young mammary skin elastic fibers were observed to be stretched perpendicular to the dermo-epidermal junction while they become gradually more fragmented and wavy with age (Fig. 7). In old cheek skin, higher amounts of elastotic tissue could be observed while collagen almost completely disappeared (right, 79-year-old cheek skin donor). Collagen (Supplementary Fig. S7) was found to be more degraded with age (from left 19-year-old mammary skin donor to middle 66-year-old mammary skin donor).

**Figure 8:**
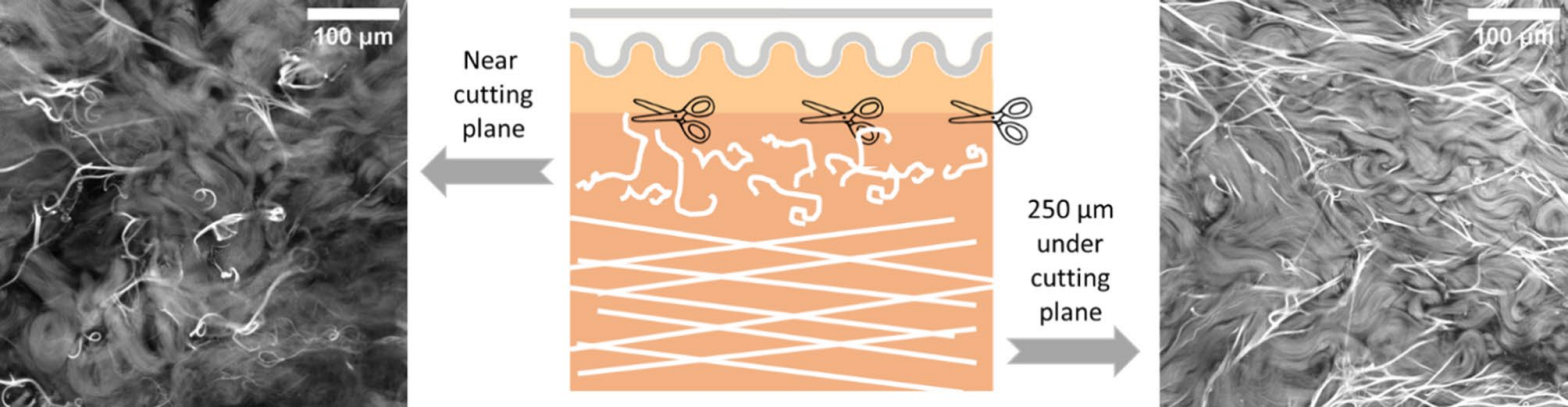
In-plane sections of optically-clarified tissue, near the cutting plane and 250 µm under the cutting plane, images obtained on the 2PEF channel of the multiphoton microscope. The elastic fibers appear with a strong fluorescence signal, the background signal is the collagen auto-fluorescence signal.

We also observed important clues pointing towards a mechanical role of the elastic network. Figure 8 shows the fluorescence signal observed on a deep dermis tissue cut in plane and thereafter optically-clarified: the elastic fibers appear straight and taut in the depth of the tissue (right), while they seem to recoil near the cutting plane. This suggests that they are under mechanical tension in the tissue and relieved of that tension near the surface by the keratome cutting process (left).

**Figure 9:**
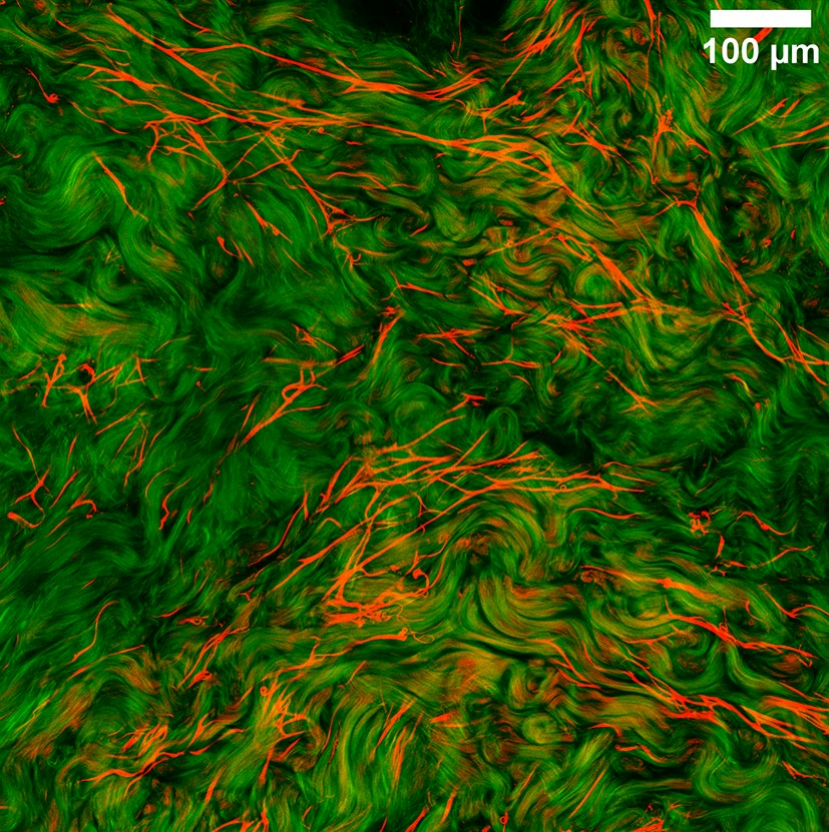
In-plane image of an optically-clarified reticular dermis sample observed in multiphoton through the 2PEF channel (red) revealing mostly elastic fibers and the SHG channel (green), revealing collagen fibers.

Furthermore, we observed that elastic fibers appeared to have a much larger typical length scale than collagen fibers. This is illustrated on Fig. 9, when observing both the SHG channel (green), revealing collagen fibers and the 2PEF channel (red), revealing mostly elastic fibers. We can see that the collagen fibers appear wavy over a relatively short range, while elastic fibers can be taut over several hundreds of microns. This effect would be even stronger when looking at the whole 3D structure and not a single optical cut (see Supplementary Video S8). This could increase the scale over which elastic fibers exert a mechanical impact on the tissue, compared to collagen fibers.

## Discussion

In this study, we report for the first time a large dataset for freshly excised human skin, and in particular facial skin, a target very relevant for cosmetics and aesthetic surgery. We present detailed layer-by-layer mechanical analysis together with micro-structural characterization. We observed significant alterations with age in skin’s mechanical properties: a significant decrease in stiffness and elastic recoil on full thickness skin explants from two different anatomical areas. In skin from the mammary area, which is also less photoexposed, we found that the onset of degradation of the mechanical properties was earlier in the superficial papillary layer than in the deeper, reticular dermis (Fig. 3).

### Comparison of results with literature

Discrepancies are found in the literature when it comes to the evolution of the skin’s stiffness with age^20^: in vivo, some studies report an increase in skin stiffness with age^21–23^ while others describe a softening^24,25^. These large discrepancies can be attributed to widely different testing conditions, and in particular type of solicitation (e.g. suction, torsion, indentation, shear waves) and amplitude. Large and small deformations can be sensitive to different elements of the dermis, meaning we effectively probe two different tissues depending on the technique used. The small amplitude shear deformations used in this study on ex vivo skin are closer to the type of deformation that shear wave elastography induces, and we found indeed that our data is in accordance with that an age-related decrease in facial skin stiffness previously reported using this technique^26^. Our observations regarding the earlier onset of ageing in the papillary dermis can be compared with the previous measurements of a sub-epidermal non-echogenic band in ultrasound imaging, which was found to be particularly well correlated with age and photoexposition^27^.

The same observation holds of course for ex vivo experiments, for which, depending on the solicitation type and amplitude, but also anatomical area, age has been shown to stiffen^28^, have no impact^13^ or soften (present study) human skin. On the other hand, the vast majority of studies that report age-related values for human skin’s elasticity (as in the recoil after a mechanical solicitation), in vivo or ex vivo and regardless of the technique, describe a degradation in elasticity, in accordance with our data^9,11,23,29^.

### Link between microstructure and mechanics

In this study, we have associated mechanical characterizations with numerous observations of the samples’ microstructure, using histological cuts stained to reveal various constituents and observations with a multiphoton microscope. We have thus acquired a large dataset, on human skin from both mammary and facial origin.

Very significant age-related changes in the dermal collagenous network were observed with both techniques. Degraded collagen may resist less to deformation (leading to a decrease of stiffness), as well as loose its ability to recoil elastically when the deformation is removed, due to damaged network connectivity. The literature suggests that the decrease in stiffness we measured for the papillary dermis may also be caused by a decrease in the diameter of collagen bundles between the ages of 20 and 60, while the collagen density remains roughly constant during the same time frame^2^.

The earlier onset of degradation of the mechanical properties in the upper dermis is to be attributed to the related changes in microstructure, shown in this study through histological data (Herovici staining (Fig. 6) and in-situ multiphoton microscopy (Fig. 7)), and confirming previous observations^5,30–32^. Indeed, Herovici staining indicates that the collagen degradation affects primarily the papillary zone in photoprotected skin. Quantification of collagen signal on Red Sirius stained histological cuts could not be correlated directly to the mechanical results (data not shown), demonstrating that collagen degradation alone cannot explain the drop in biomechanical properties, in particular, the decrease in the elastic recoil of aged skin and even more so for the photoexposed skin.

For most of our donors, the storage modulus of full thickness skin was larger than that of each of the two dermal layers, papillary or reticular. One explanation for this could be that the epidermis, which is present on our full thickness samples and removed from our dermal sublayer samples, plays a significant role in the mechanical properties of the skin. It is however unlikely given the composition of the epidermis (living cells and very stiff but extremely thin cornified layer), and the epidermal contribution is often considered negligible compared to the dermis^28^. The second possible explanation is that the preparation of the dermal sublayer (keratome cutting and chemical treatment to remove the epidermis) damages considerably the tissue, impacting the mechanical properties. No tissue damage was observed on histological cuts of upper and deeper layers (see Supplementary Fig. S2), so this damage is likely to be relatively limited and localized. Finally, another likely explanation is that the inter-layer cohesion could play a major role in skin biomechanics, in particular at the reticular/papillary interface and at the dermo-epidermal junction. The removal of this cohesion by the cutting process may explain the degraded mechanical properties.

Our histological and multiphoton data also provide clues to a potential important role of elastin and fibrillin organization in the papillary zone. We observed indeed with the multiphoton that the elastic fibers appear taut in the depth of the tissue and seem to recoil near the surface where they were cut by sample preparation (keratome). This observation is reminiscent of a rubber band or a spring which narrows and coils when cut, suggesting a mechanical tension in the elastic fibers in the original state. Skin is known to be naturally stretched in vivo, following the well-known Langer lines, and that pretension has been observed to decrease with age^10^. Interestingly, this appears to be the case even though we manipulate here ex vivo skin, for which much pretension has been removed during sample excision. This hints that the elastic fibers might have been even more tensed in vivo. An interesting follow-up study might be to try to quantify the fiber tension by using the two-photon laser to physically cut the fibers in situ. The relevance of that mechanical tension in the elastic fibers might be accentuated even further by the fact that the elastic fibers appear to span over relatively long length scales (several hundreds of microns) in mosaic multiphoton images with a large field of view (Fig. 9). Again, it is remarkable that this is observed in excised ex vivo skin and suggests that the elastic fibers might extend even further in vivo. A direct observation could also be possible in vivo, but imaging the deeper dermis remains a challenge due to light scattering in the epidermis.

Notably, on histological sections after orcein staining, we observed an age-related degradation of the vertical elastic network (oxytalan/eulanin), with a similar aggregation (Supplementary Fig. S6). Thus, we can draw an analogy between the cutting process of the keratome in our study and the effect of enzymes with age, for example elastase and certain MMPs such as MMP9 or MMP12, which have elastolytic activity^33,34^. The gradual recoil of elastic fibers from taut candelabra structures in young photoprotected skin to wavy fragments in photoexposed aged skin suggests that age and photoexposition may have a similar impact on the structure, and thus mechanics, of elastic fibers than a keratome cut, albeit at a much longer time scale.

Anatomically, the epidermis and reticular dermis are separated by the subpapillary plexus. The papillary dermis contains thinner, sparsely arranged collagen and oxytalan fibrils partially oriented perpendicular to the dermo-epidermal junction, whereas reticular dermis is dominated by thicker and denser collagen bundles with some preference for orientation parallel to skin surface and interspersed with elastic fibers. Our multiphoton data from thin transversal sections of skin (Fig. 7) shows how the elastin/oxytalan network becomes degraded with age, progressively becoming shorter, fragmented and distorted.

As this elastic network ensures the cohesion between the different cutaneous layers, in this case reticular dermis with epidermis via the dermo-epidermal junction, it is probable that they play a very important role in skin’s mechanics and become, with age, some of skin’s mechanical “weak links”^35^. The branching morphology and tautness of elastic fibers in the papillary dermis appear to ensure the optimal connectivity and mechanical coupling between the stiffer reticular dermis and the softer epidermis, and may also be crucial to support the undulations of the dermo-epidermal junction, all of which are crucially degraded with age. Therefore, fibrillin-elastin assembly (and stability) seems to be a particularly suitable target for cosmetic treatments (including fibulin 5^36^ and other factors).

Despite the size of the database acquired, the intra and inter-individual variability remained too large to allow for a full statistical analysis. One explanation for this might be that the mechanical properties are measured on the full sample, at a large length scale (several millimeters), while the microstructure was characterized using histological sections, which only give a partial view of the full sample. A better approach might be to use successive histological cuts (a very time-consuming process), or better yet microscopy images on optically clarified tissue. Both these approaches would have to be associated with appropriate image quantification to handle these complex images. Additionally, and particularly in the case of facial skin, it seems very probable that the skin appendices (glands, hairs) would influence the skin mechanical properties. Finally, it might be necessary to associate the microstructure quantification (quantity and structure) with some component quality characterization (e.g. the ability of proteoglycans to retain water, the collagen maturity) to get a full picture on the link between dermal mechanics and matrix composition.

### Relevance for biology

#### Impact of dermis softening on fibroblast activity

Our results are relevant from a biological perspective to explain other phenomena observed in aged skin. Firstly, it seems very probable that the softening of the dermis will affect the primary occupants of the dermis, namely the fibroblasts. It has been shown that fibroblasts in aged skin tended to attach less to the fragmented collagen fibers and have a reduced cell spreading compared to young skin^37,38^. As fibroblasts are very mechano-sensitive cells, this was thought to lead to a decreased mechanical stimulation of the cells and in turn decreased matrix synthesis^39^. A softer dermis could make the cell attachment even more difficult, leading to a continued decrease in mechanical stimulation and matrix production.

#### Possible impact of papillary dermis softening on sensory mechanoreceptors

We also think the softening of the dermis, papillary in particular, can have an impact on other mechano-sensitive structures in the dermis, namely the mechanoreceptors responsible for tactile perception. Decreased mechanical properties of the dermis may lead to a decreased mechanical coupling between the matrix and mechanoreceptors, and thus be partly responsible for the decreased tactile acuity observed in elderly populations^40^.

#### Possible impact of papillary dermis softening on DEJ and epidermis

Finally, if we look beyond the dermal compartment, the softening of the dermis, in particular the upper layer, could be a possible mechanical factor in the flattening of the dermo-epidermal junction, responsible for a decrease in nutrient supply to the epidermis and altered epidermal renewal^34^. Furthermore, it is now well established that cells, including fibroblasts, can feel their mechanical environment through mechanobiological pathways and that this in turn influences their behavior. Yet, it has been shown in previous studies that the fibroblasts do influence the quality of the epidermal structures, through their sheer presence^41^ as well as phenotype (e.g. aged or young^42^). Hence, an altered upper dermal layer could impact the epidermal renewal process and thus contribute to the skin’s ageing process. In turn, a sub-optimal epidermal organization might impact the dermal remodeling, and thus mechanical properties (as has been demonstrated recently on vitro reconstructed skin models), again leading to a downward cycle.

Further studies on vitro reconstructed skin models could be useful to investigate the results we obtained in ex vivo skin samples: their structure and mechanics remains far from actual skin, but their simplicity means specific targets can be altered to quantify the impact in the mechanics.

### Relevance for ageing perception

Finally, our study is particularly relevant to explain the age-related changes in skin perception with age, in particular perceptions of firmness loss or, as reported by consumers, “plumpness” (elastic recoil observed after indenting the cheek with one’s finger) loss. Indeed, these aspects, whose relevance for actual perception of age has recently been confirmed^43^, may be tightly linked to the mechanical properties of skin and in particular the dermal compartment.

Extreme spatial variability in the microstructure was observed on skin from facial origin. We believe that many important hallmarks of ageing related to unevenness (e.g. surface topography, tone, micro relief, “orange peel” appearance) are likely the result of this age-related increase in heterogeneity of internal dermis structures. Indeed, facial appearance is intimately linked with the perception of age and health, where the skin surface condition plays a major role^44^. Any blemish or imperfection can degrade the perception of facial youthfulness and health, such as spots, or color heterogeneities, acne lesions and scars, dilated pores, folds and wrinkles. Many of these imperfections have their origin in deeper skin layers. For example, dilated skin pores have been shown to result from reduction of sweat gland volume^45^. Similarly, fine lines and wrinkles appear and propagate where the dermis structure is affected^46^. Our results demonstrate that structural heterogeneity in aged facial skin is intrinsically very high, and only increases with age (see Supplementary Fig. S4), which is likely to contribute to degraded skin appearance.

Improving the microstructural homogeneity may be an opportunity for future cosmetic or surgical treatments aiming to improve facial skin appearance in a curative mode, by targeting specifically the damaged areas (either directly or through cell-mediated remodeling). Of course, our work also demonstrates that it is most important to protect one’s skin from an early age, preferably before 40 years old. In both cases, the current study points to the upper, papillary dermis, as a very relevant target to cure or prevent the effect of age on dermal mechanics, and thus preserve skin overall youthful appearance and feel.

## Materials and methods

### Ex vivo skin samples

Fresh human ex vivo skin samples from two anatomical locations were used: mammary skin (photoprotected) and cheek skin (photoexposed). Skin samples from the photoprotected mammary area were obtained after breast surgeries from 3 suppliers (Alphenyx, Marseille, France; Biopredic, Saint-Grégoire, France; Sterlab, Vallauris, France). Skin samples from the photoexposed cheek area were obtained after lifting surgeries (Icelltiss, Toulouse, France), from the outermost area of the cheek (closest to the ear). All skin samples were from Caucasian type donors (Fitzpatrick skin types I to III).

Written informed consent was obtained from the donors according to the principles expressed in the Declaration of Helsinki and in article L.1243-4 of the French Public Health Code. Patients’ written informed consents were collected and kept by the surgeon. The samples were anonymized before their reception by the authors. Only age, sex and anatomical site of samples were specified to the authors. The authors did not participate in sample collection. Given its special nature, surgical residue is subject to specific legislation included in the French Code of Public Health (anonymity, gratuity, sanitary/safety rules and no publicity for donation). This legislation does not require prior authorization by an ethics committee for sampling or use of surgical waste.

**Figure 10:**
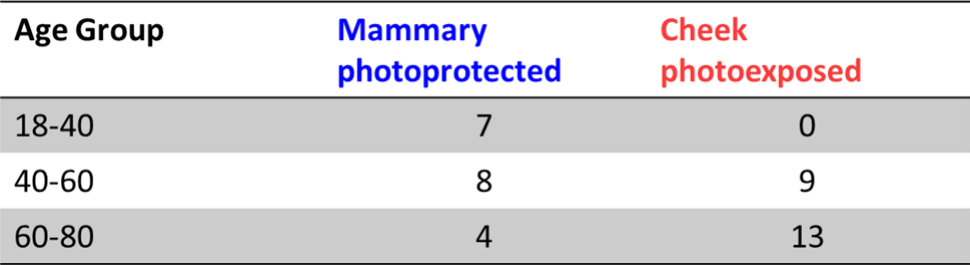
Number of donors per age group and type of sample.

Samples from a total of 41 donors were considered: 19 for mammary skin (all female donors) and 22 for cheek skin (20 female donors and 2 male donors). The donors were grouped by 20-year age groups to obtain a more interpretable representation of the data. The number of samples per age group is represented in Fig. 10.

Fragments of full-thickness skin, including subcutaneous fatty tissue, were shipped non-frozen, on a gauze soaked in culture medium and packed in a polystyrene box with cold accumulators. They were delivered on the day following surgery and the experiments performed within 24 h. Before any experiment, the fat was carefully removed with scissors, taking care to preserve the dermis from damage.

### Keratome—skin in-plane cutting

In photoprotected mammary skin, we used a keratome (Aesculap-Wagner, micro, Braun Medical SAS, Boulogne-Billancourt, France) to split the tissue roughly at the interface of the two dermal sublayers: papillary dermis (the upper 0.3 mm of tissue, with respect to the surface) and reticular dermis (deeper layer). Such splitting was not possible on photoexposed cheek skin due to small sample size.

**Figure 11:**
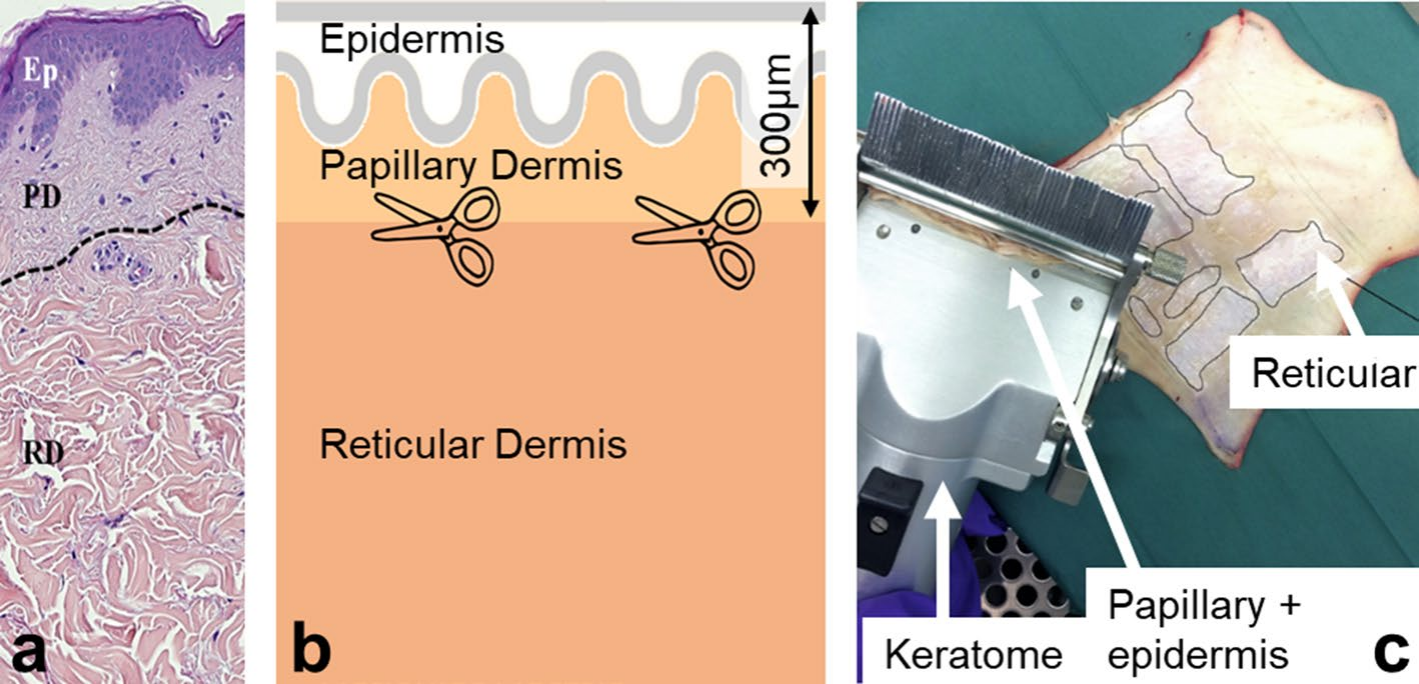
Splitting excised skin into two sub-layers using the keratome. (**a**) Histological section of normal human skin showing papillary (PD) and reticular (RD) dermis sub-layers (separated by a dotted line for clarity). *Ep* epidermis. Keratome separation at 300 µm roughly divides the two sub-layers (**b**). Keratomized areas on a skin sample (**c**) are shown with thin black outlines.

Keratome is a tool for cutting horizontal layers in tissues of defined thickness with a sharp moving blade. The keratome was set to cut at 0.3 mm into the skin in order to separate the two dermis layers. The keratome cutting process is depicted in Fig. 11.

The upper part contains the epidermis and the papillary dermis. The lower part includes the reticular dermis and possible remains of the hypodermis layer. Due to inevitable variability of skin samples, keratome handling and the skin not being plane, it was impossible to guarantee that the separation occurred exactly at the interface between the papillary and reticular sublayers. However, histological verification on cross-sections of full thickness, upper and deeper layers from the same donor confirmed that the upper part consisted mostly of papillary dermis with some reticular dermis, while the deeper part consisted only of reticular dermis (see Supplementary Fig. S9).

### Epidermis removal

Among different epidermal/dermal separation techniques, we have selected an ammonium thiocyanate treatment to remove the epidermis without damaging the dermis^47^. A 30-min treatment with 3.6% ammonium thiocyanate (Sigma, Saint-Quentin Fallavier, France) in Hank's Balanced Salt Solution (HBSS HyClone, GE Life Sciences, Logan, USA) was applied to both upper and deeper layers. HBSS is a saline solution known to limit tissue swelling by maintaining optimum physiological pH. After treatment, the epidermis was easily detached from the papillary dermis using tweezers. All samples were placed in HBSS before rheometer measurements. At the end of this process, we obtained upper (mostly papillary dermis) layers without epidermis, which we further refer to as “Upper Dermis” samples, and reticular dermis referred to as “Deep Dermis” samples. Prior to mechanical testing, the samples remained at room temperature (22–25 °C) for at least an hour to equilibrate.

### Mechanical measurements

We used a Kinexus Pro + rheometer (Malvern Panalytical SAS, Orsay, France) to perform mechanical characterization of the samples. The rheometer was used in the parallel-plate configuration, with sanded plate surfaces. The upper plate diameter was 8 mm and the lower one 50 mm. Skin samples were cut to approximately 12 mm disks, largely exceeding the 8-mm circular area covered by the upper plate. The plates were temperature-controlled at 25 °C, and HBSS was added to limit sample dehydration. In a typical test, the upper plate of the rheometer slowly descends on the sample until reaching a normal force of 0.05 N and stabilizes for 10 s. The preload prevents the sample from slipping. The gap between plates is then set and a dynamic amplitude sweep is carried out from 0.01 to 0.1% deformation (with regards to sample thickness) applied at a frequency of 1 Hz. The outputs of the experiments are the storage modulus G′ (stiffness) and the phase angle δ (relative importance of elastic and viscous behaviors).

Additionally, creep and recovery tests were performed by imposing a compressive deformation using the same instrument and plate configuration as above. In a typical creep and recovery test, 0.1 N of compressive force is applied for 300 s, then the stress is abruptly reduced to 0.05 N and the recovery in the gap is followed for 500 s. The compressive stress is not removed entirely to make sure that the contact between sample and plate is maintained continuously. A typical strain vs time curve is presented in Fig. 2, upper panel.

We define a quantitative measure of immediate elastic rebound of the tissue, which is inspired by the net elasticity parameter used to describe the results of in vivo measurements of skin elasticity for clinical instruments such as the Dermal Torque Meter (Monaderm, Monaco) or Cutometer (Courage + Khazaka electronic GmbH, Köln, Germany)^11^. For both the creep and recovery phases, we define the immediate elastic response as the deformation after 5 s, which is the time needed for deformation stabilization by the rheometer. Then, we define Ue as the compressive strain experienced by the sample 5 s after application of the 0.1 N compressive stress (creep phase) and Ur as the strain drop over the first 5 s following the reduction of compressive stress to 0.05 N (recovery phase). The net elasticity is calculated by the ratio Ue/Ur. Other ways to quantify the sample elasticity are shown in the supplementary data (Supplementary Fig. S1).

### Histological observations

Skin sections perpendicular to the skin surface were performed close to the samples used for mechanical measurements. The sections were fixed in neutral formalin 4% then embedded in paraffin. Paraffin sections (5 µm) were processed before specific colorations. Five stainings were performed: (I) Hematoxylin Eosin Safron (HES) for the global morphological aspect, (II) Orcein stain to visualize the elastic fibers network, (III) Sirius Red stain to observe the collagen network, (IV) Herovici stain that distinguishes mature (colored purple) and immature or degraded collagen (colored blue), (V) Mowry Van Gieson stain to observe collagen network (colored purple) and proteoglycans (colored blue-green). The sections were scanned using NanoZoomer-HT-C9600-12 (Hamamatsu Photonics, Iwata City, Japan). Quantifications of the collagen and elastic networks present in the dermal part were carried out using the Fiji software: the number of pixels per unit area in the color related to each coloration was quantified on each sample, over a depth of 500 μm for the total dermis and 200 μm for the papillary dermis. The thickness of the epidermis was measured with the NDPview software taking into consideration only the living layers (without the stratum corneum).

### Multiphoton images

Skin samples were imaged using a commercial Leica SP8 multiphoton microscope with FLUOTOR 25 × 0.95 NA objective (Leica) coupled to an infrared pulsed laser at 760 nm wavelength (Coherent Chameleon Ultra II laser). Multiphoton images were composed of two nonlinear signals without any labeling required: second-harmonic generation (SHG) and two-photon excited fluorescence (2PEF). SHG occurs only for noncentrosymmetric structures. In the skin, this signal is specific only to fibrillar collagen and 2PEF reveals auto-fluorescent compounds such as cells and elastic fibers (collagen also has a weak auto-fluorescence). Both signals were collected by the same objective used for the excitation and separate by a dichroic filter (Semrock FF409-Di03-25x36). The 2PEF signal was collected between 409 and 680 nm (Semrock FF02-409/LP-25) and the SHG signal was collected at 380 nm ± 7 nm (Semrock FF01-380/14-25).

Images parallel to the skin plane were acquired after optical clarification of the samples. The samples were first fixed samples in neutral formalin 4%, then clarified by immersing them in RapidCLear 1.52 (Sunjin Lab) medium for 48 h. Finally, the samples were put between a glass slide and a coverslip with a 1 mm Press-To-Seal Silicone Isolator spacer (Grace Bio-Labs), with medium in between.

For cross-sections, the samples were first included in embedding medium Tissue-Tek and frozen on a dry ice and ethanol bath. Then they were cut into 50 µm cross-sections with cryomicrotome at − 30 °C. Cross-sections were placed between microscope slide and coverslip with montage medium Dako.

### Statistics

Data are presented as boxplots, generated using OriginPro 2019 (OriginLab, Northampton, USA). Each point represents one skin sample. Multiple skin samples were acquired per donor. The bold line is the median, the box limits are the 1st and 3rd quartiles. The significance tests were performed with the R software (R Core Team, 2014) using the nonparametric Wilcoxon test, appropriate for the number of samples considered.

## Supplementary Information

Supplementary Figures.

Supplementary Video S8.
