# Supplementary material for: A mechanistic view on the aging human skin through ex vivo layer-by-layer analysis of mechanics and microstructure of facial and mammary dermis
Source: Sci Rep. 2022 Jan 17;12:849. doi: 10.1038/s41598-022-04767-1 (PMC8764052; doi:10.1038/s41598-022-04767-1)
Supplement: Supplementary file 1 — Supplementary Figures. [file 41598_2022_4767_MOESM1_ESM.docx]

# **Supplementary Material**


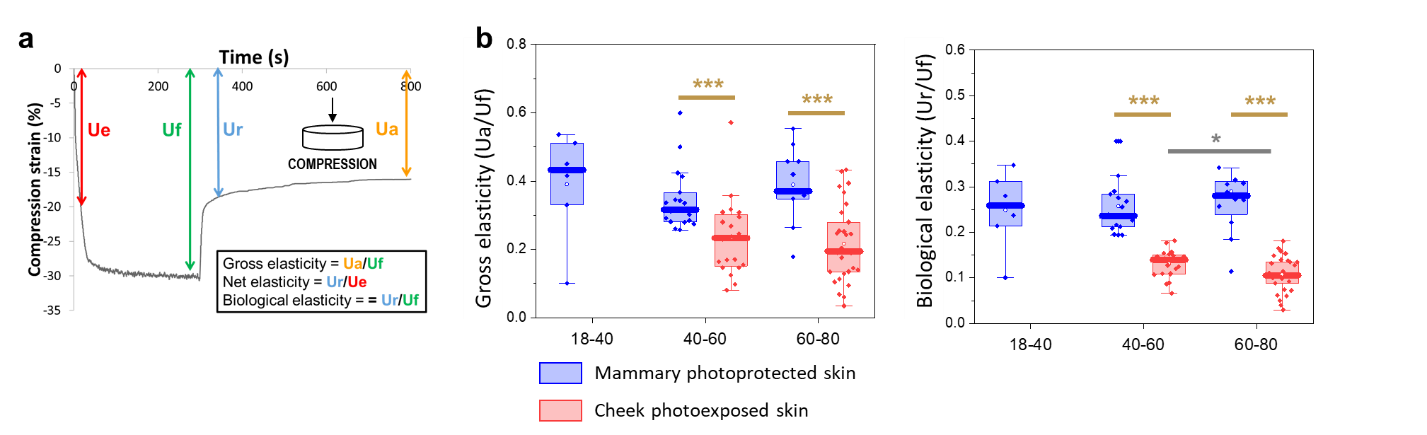


Supplementary figure S1: Elastic recoil of full-thickness skin after compressive deformation perpendicular to skin surface. (a) Definition of parameters Ue, Uf, Ur and Ua, along with the gross, net and biological elasticity parameters (b) Gross (Ua/Uf) and Biological (Ur/Uf) elasticity of photoprotected mammary (blue) and photoexposed cheek (red) skin as a function of age group. One dot represents one skin sample. Multiple samples were obtained for each donor. * p-value <0.05, ** p-value <0.01.


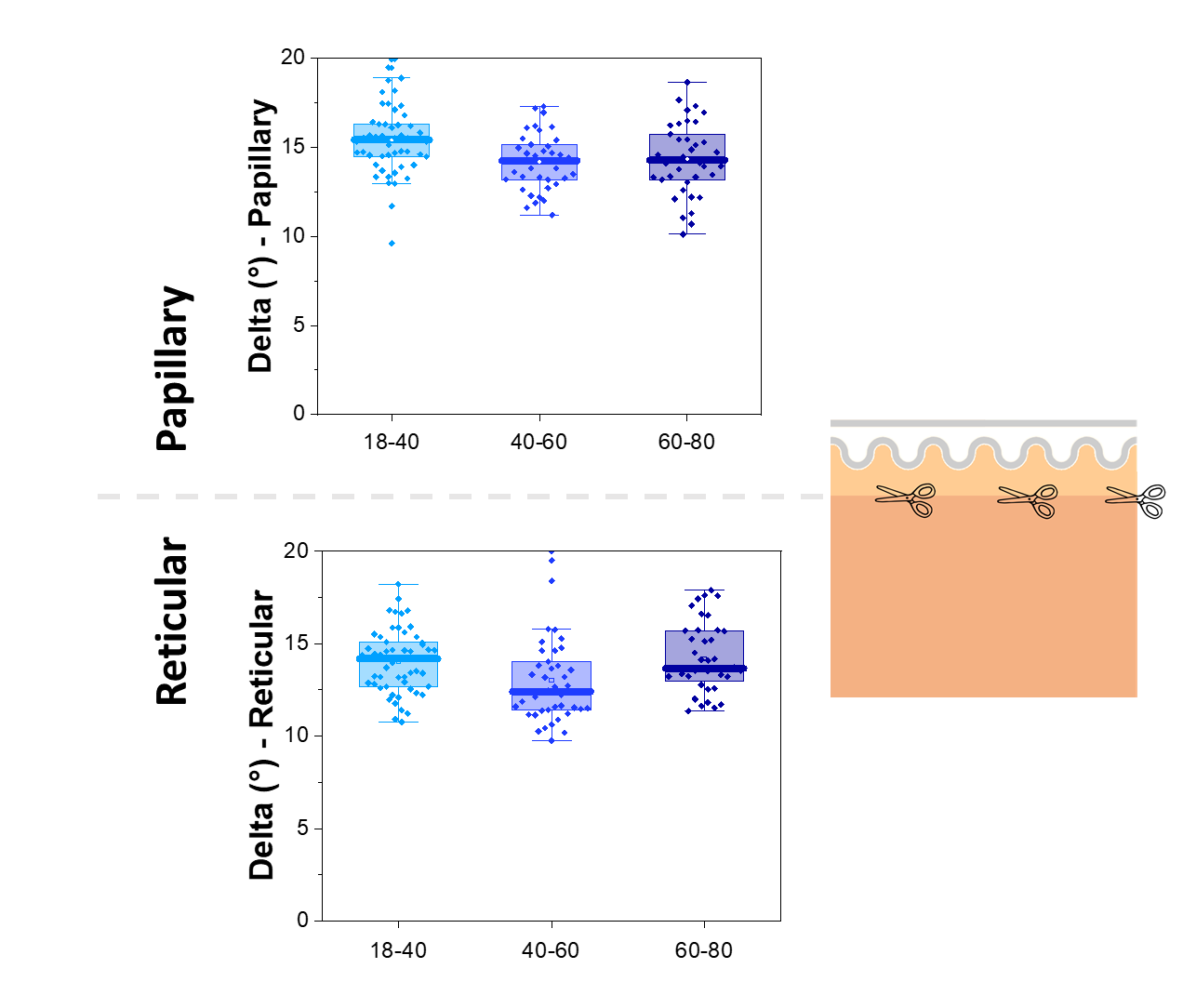


Supplementary figure S2: Phase angle delta (°) as a function of age group for the papillary dermis (top) and reticular dermis (bottom) for mammary skin. The epidermis layer has been removed prior to measurements. One dot represents one skin sample. Multiple samples were obtained for each donor.


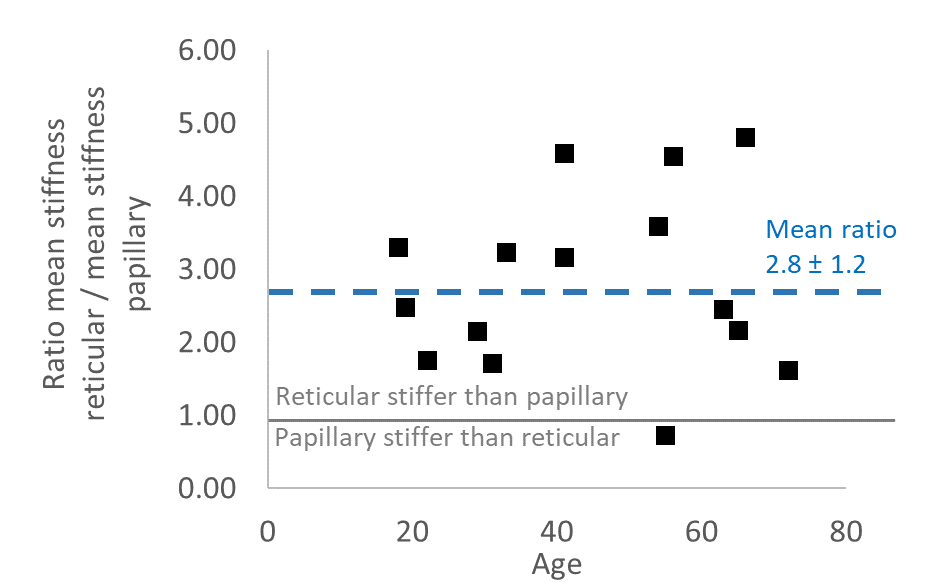


Supplementary figure S3: Ratio of mean storage modulus G’ for the upper dermis over mean storage modulus G’ for the deeper dermis, for all donors


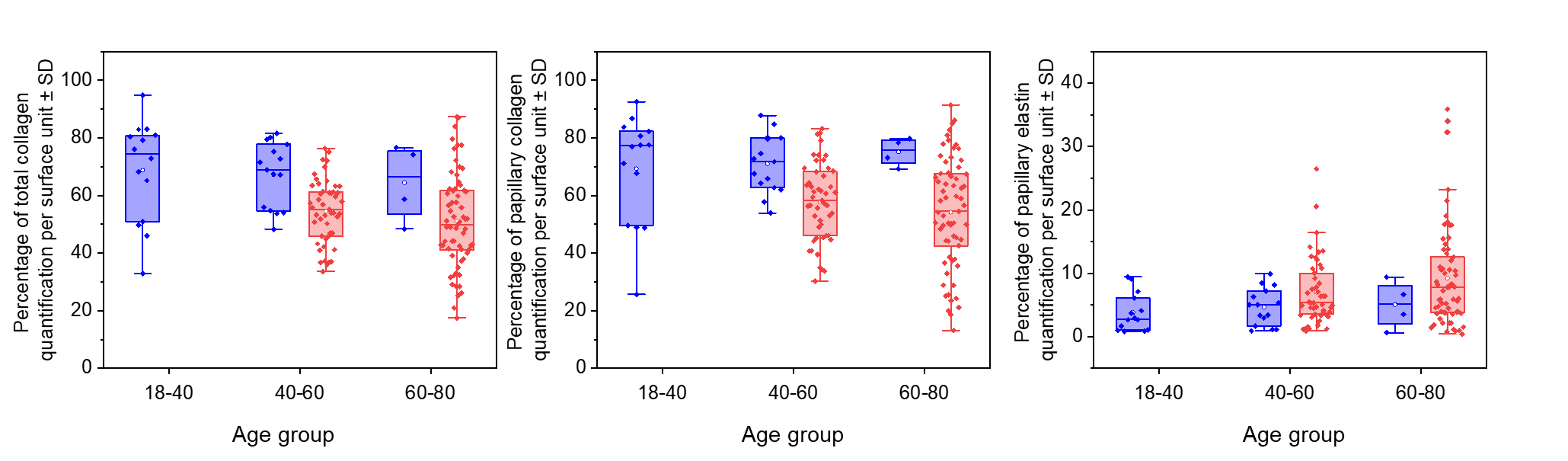


Supplementary figure S4: Quantification (in percentage per surface unit) of total collagen, papillary collagen or papillary elastin from mammary (blue box plot) or cheek (red box plot) skin in function of age group.


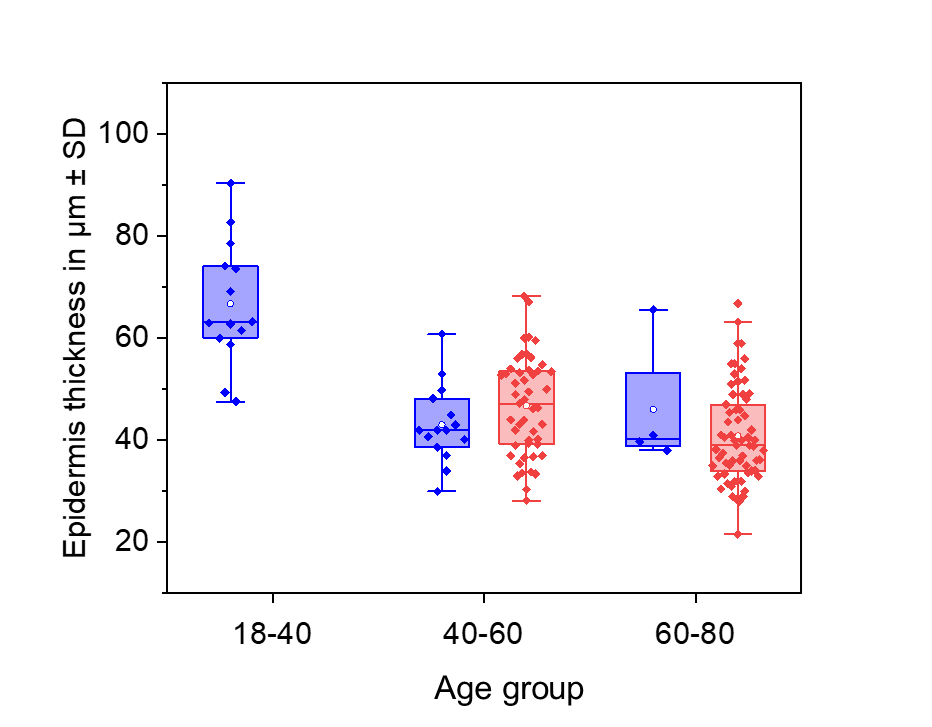


Supplementary figure S5: Measurement of Epidermis thickness from mammary (blue box plot) or cheek (red box plot) skin in function of age group.


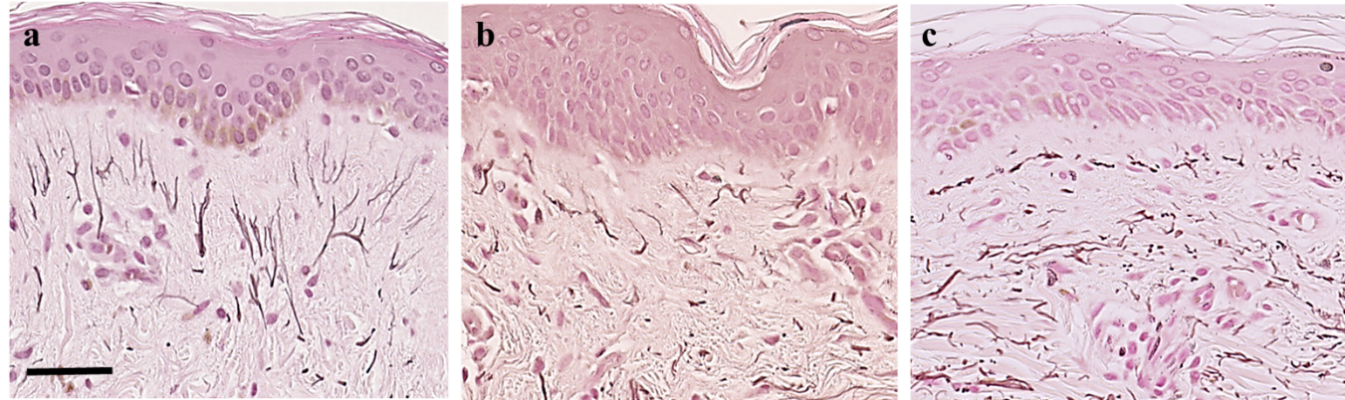


Supplementary figure S6: Elastin network by histological Orcein coloration from mammary from young (a) and old (b) donors or cheek (c) human skin. Scale is 50 µm.


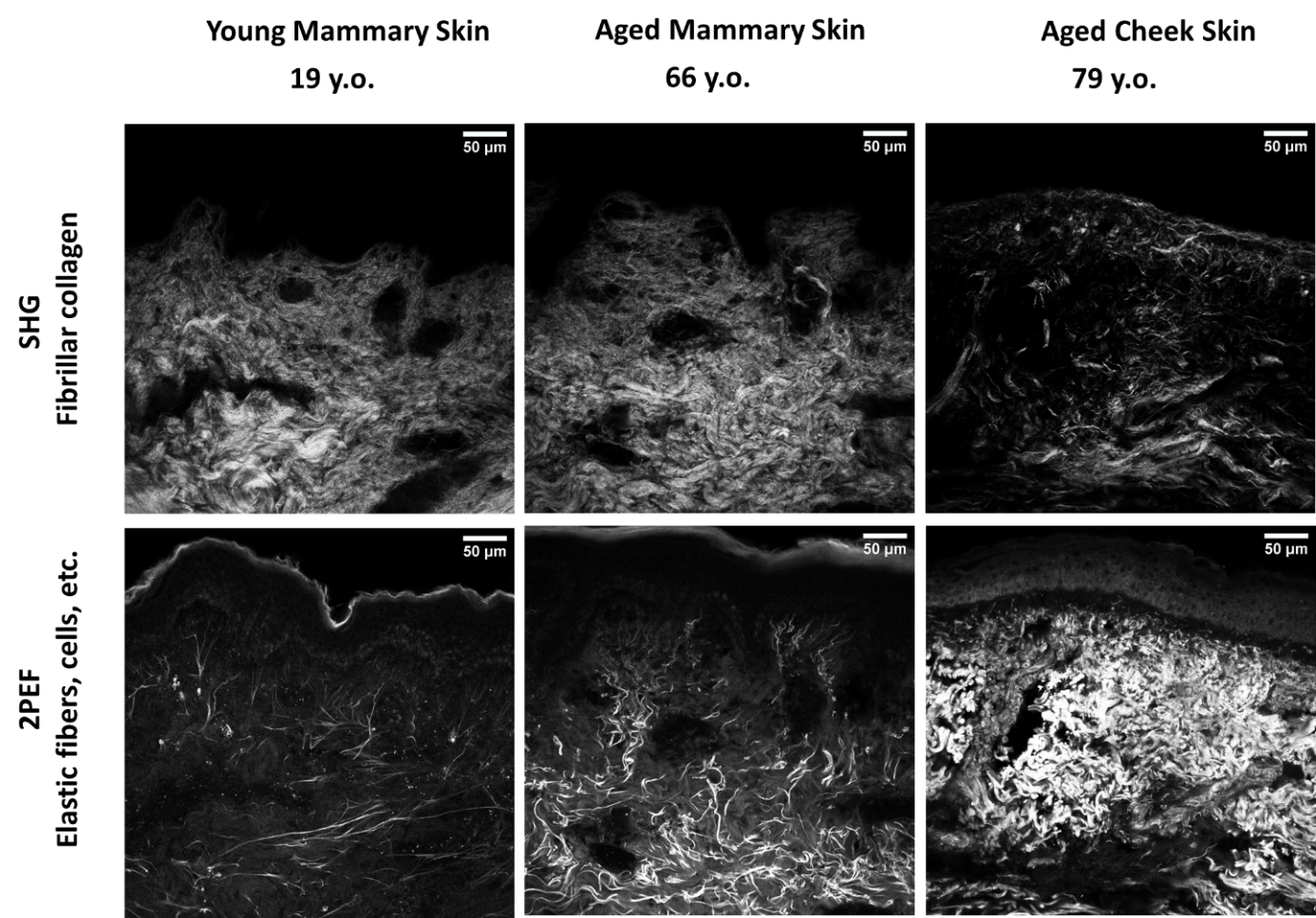


Supplementary figure S7: Second Harmonic Generation (SHG) images revealing collagen fibers in cross-sections of young mammary skin (19 years old), old mammary skin (66 years old) and old cheek skin (79 years old) observed in multiphoton microscopy.

Supplementary video S8: In-plane stack of images from an optically-clarified dermis sample observed in multiphoton through the 2PEF channel (red) revealing mostly elastic fibers and the SHG channel (green), revealing collagen fibers. The full dermis depth is imaged (1200µm). Field of view: 880 µm x 880 µm.


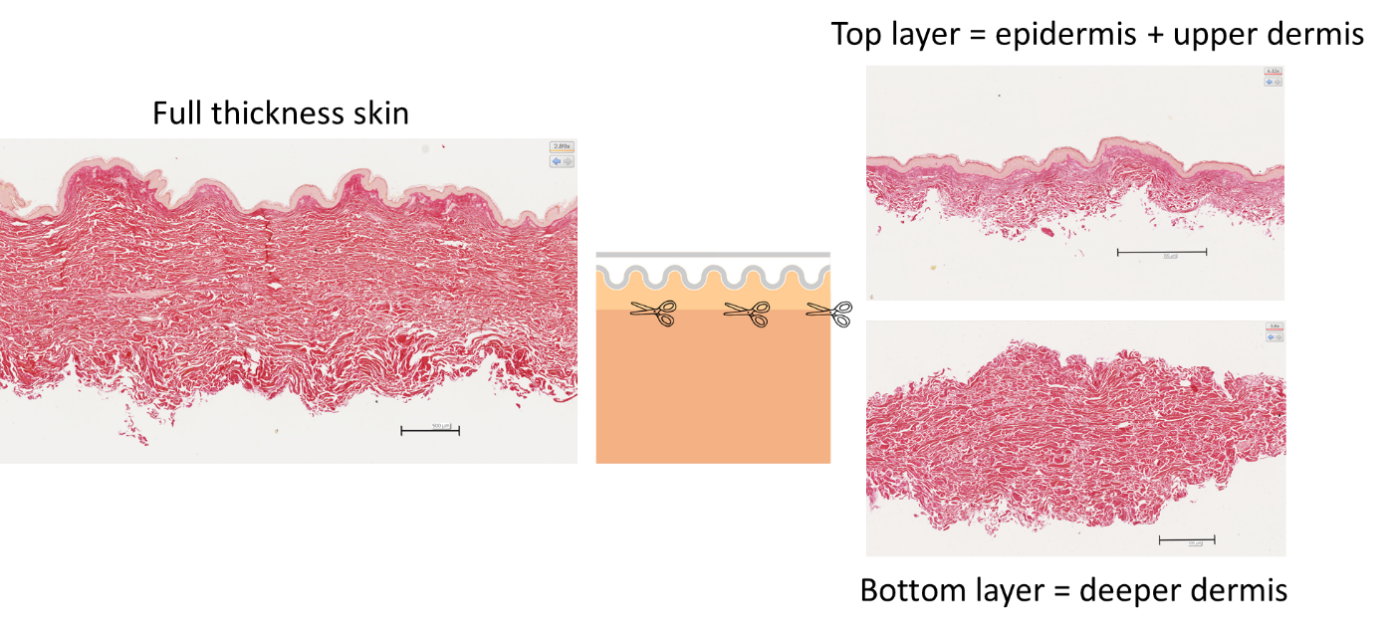


Supplementary figure S9: Histological cross-sections stained with Sirius Red of a full thickness sample (left) and separated dermal layers: top layer containing epidermis and upper dermis (top right), and bottom layer consisting of the deeper dermis (bottom right).

Supplementary figure S1: Elastic recoil of full-thickness skin after compressive deformation perpendicular to skin surface. (a) Definition of parameters Ue, Uf, Ur and Ua, along with the gross, net and biological elasticity parameters (b) Gross (Ua/Uf) and Biological (Ur/Uf) elasticity of photoprotected mammary (blue) and photoexposed cheek (red) skin as a function of age group. One dot represents one skin sample. Multiple samples were obtained for each donor. * p-value <0.05, ** p-value <0.01.

Supplementary figure S2: Phase angle delta (°) as a function of age group for the papillary dermis (top) and reticular dermis (bottom) for mammary skin. One dot represents one skin sample. Multiple samples were obtained for each donor.

Supplementary figure S3: Ratio of mean storage modulus G’ for the upper dermis over mean storage modulus G’ for the deeper dermis, for all donors

Supplementary figure S4: Quantification (in percentage per surface unit) of total collagen, papillary collagen or papillary elastin from mammary (blue box plot) or cheek (red box plot) skin in function of age group.

Supplementary figure S5: Measurement of Epidermis thickness from mammary (blue box plot) or cheek (red box plot) skin in function of age group.

Supplementary figure S6: Elastin network by histological Orcein coloration from mammary from young (A) and old (B) donors or Cheek (​photoexposed) (C) human skin. Scale is 50 µm.

Supplementary figure S7: Second Harmonic Generation (SHG) images revealing collagen fibers in cross-sections of young mammary skin (19 years old), old mammary skin (66 years old) and old cheek skin (79 years old) observed in multiphoton microscopy.

Supplementary video S7: In-plane stack of images from an optically-clarified dermis sample observed in multiphoton through the 2PEF channel (red) revealing mostly elastic fibers and the SHG channel (green), revealing collagen fibers. The full dermis depth is imaged (1200µm). Field of view: 880 µm x 880 µm.

Supplementary figure S9: Histological cross-sections stained with Sirius Red of a full thickness sample (left) and separated dermal layers: top layer containing epidermis and upper dermis (top right), and bottom layer consisting of the deeper dermis (bottom right).
